# New autoantibodies in early rheumatoid arthritis

**DOI:** 10.1186/ar4255

**Published:** 2013-07-25

**Authors:** Caroline Charpin, Fanny Arnoux, Marielle Martin, Eric Toussirot, Nathalie Lambert, Nathalie Balandraud, Daniel Wendling, Elisabeth Diot, Jean Roudier, Isabelle Auger

**Affiliations:** 1 INSERM UMRs 1097, Aix Marseille Université, 163 Avenue de Luminy, 13288 Marseille, France; 2 Rheumatology, IML, APHM, 270 Boulevard de Sainte Marguerite, 13009 Marseille, France; 3 Service de Rhumatologie, CHU Jean Minjoz, 2 Boulevard Fleming, 25030 Besançon, France; 4 Service de Médecine Interne, CHU Bretonneau, 2 Boulevard Tonnellé, 37000 Tours, France

**Keywords:** rheumatoid arthritis, autoantibodies, early biomarkers

## Abstract

**Introduction:**

Rheumatoid arthritis (RA) is a chronic inflammatory joint disease causing articular cartilage and bone destruction. Since irreversible joint destruction can be prevented by intervention at the early stages of disease, early diagnosis of RA is important. In this study, we identified new autoantibodies in the sera of patients with early (less than one year) RA.

**Methods:**

We screened the sera of 20 RA patients with disease duration less than one year, 19 RA patients with disease duration more than five years and 23 controls on 8,268 human protein arrays. We confirmed the validity of protein array detection by ELISA assays. We then performed epitope mapping with overlapping 15-mers to analyze RA sera reactivity.

**Results:**

WIBG (within BGCN homolog (Drosophila)), GABARAPL2 (GABA(A) receptor associated protein like 2) and ZNF706 (zinc finger protein 706) proteins are preferentially recognized by autoantibodies from early RA patients. Of interest, autoantibodies to WIBG are very specific for early RA. Indeed, 33% of early RA patients' sera recognize WIBG versus 5% of RA patients with disease duration more than 5 years and 2% of controls. We identified three linear peptides on WIBG GABARAPL2 and ZNF706 that are preferentially recognized by sera of early RA patients.

**Conclusions:**

We identified new autoantibodies associated with RA with disease duration less than one year. These autoantibodies could be used as diagnosis markers in RA patients.

## Introduction

Rheumatoid arthritis (RA) is a chronic autoimmune disease affecting 0.5% of the world population. It is characterized by inflammation of joints that results in cartilage and bone destruction, joint deformity and loss of mobility. Although RA has been extensively studied, its cause is unknown. Treatment is directed towards reducing inflammation and stopping joint destruction. Since joint destruction can be stopped by intervention at the early stages of the disease, early diagnosis of RA is important. However, diagnosis of RA can be difficult. Immunologic tests that can be performed for the diagnosis of RA include detection of anti-citrullinated protein antibodies (ACPA) [1]. ACPA identify 65% of RA patients. Negative ACPA testing does not exclude RA.

To identify new autoantibodies in RA, we selected sera from 20 RA patients with disease duration less than one year, 19 RA patients with disease duration more than five years and 23 controls, to screen 8,268 human protein arrays. We identified 25 autoantigens recognized by the sera of early RA patients.

To confirm the validity of protein array detection, we used the 25 purified proteins in ELISAs. We tested the sera of 124 RA patients with disease duration less than 1 year and 40 RA patients with disease duration more than 5 years. We also tested 186 controls (81 patients with ankylosing spondylitis (AS), 30 patients with psoriatic arthritis (PsA), 19 patients with systemic lupus erythematosus (SLE), 16 patients with systemic sclerosis (SSc) and 40 healthy subjects). We validated three proteins that are significantly recognized by autoantibodies from patients with early RA. These proteins are: within BGCN homolog (*Drosophila*) (WIBG), GABA(A) receptor-associated protein-like 2 (GABARAPL2) and zinc finger protein (ZNF706). Of interest, autoantibodies to WIBG are very specific for early RA.

Epitope mapping on WIBG, GABARAPL2 and ZNF706 allowed us to identify peptide targets of autoantibodies, which may prove interesting in the diagnosis of early RA.

## Materials and methods

### Patients and controls

Informed consent was obtained from all patients and controls. The study protocol was approved by the Ethics Committee of Marseille, France (DC2008-327). The characteristics of patients and controls are shown in Table 1. ACPA were detected using the anti-CCP2 kit from Eurodiagnostica (Malmö, Sweden) for all RA patients and controls. Rheumatoid factor (RF) was detected by ELISA for all RA patients using the Orgentec Kit (Mainz, Germany).

**Table 1:** Patients and controls for protein arrays and ELISA

	Number	Age at diagnosis	Disease duration	Anti-CCP-positive	RF-positive
		(years)	(years)	(%)	(%)
					
RA <1 year	144	54	0.8	70	68
					
RA >5 years	59	48	11	87	75
					
AS	88	34	10	1	nd
					
PsA	30	41	6	3	nd
					
SLE	21	37	8	5	nd
					
SSC	20	51	0.5	5	nd
					
Healthy	50	nd	nd	0	nd

ELISA, enzyme-linked immunosorbent assay; CCP, cyclic citrullinated peptide; RF, rheumatoid factor; RA, rheumatoid arthritis; AS, ankylosing spondylitis; PsA, psoriatic arthritis; SLE, systemic lupus erythematosus; SSC, systemic sclerosis; nd, not determined.

### Serum samples for protein arrays

We analyzed the sera of 39 RA patients from the rheumatology unit at Sainte Marguerite Hospital in Marseille. Twenty RA patients had disease duration less than one year and 19 more than five years (time elapsed since first diagnosis by a physician). All RA patients fulfilled the 2010 American College of Rheumatology (ACR)/European League Against Rheumatism (EULAR) revised criteria [2]. Controls were seven patients with AS, two with SLE and four with SSc, from the rheumatology unit at Sainte Marguerite Hospital in Marseille. Ten healthy controls were recruited among laboratory staff volunteers.

### Protein arrays

We used Invitrogen (Carlsbad, California, USA) ProtoArrays that contain 8,268 human proteins. All proteins have been expressed as glutathione-S-transferase (GST) fusion proteins, purified under native conditions and spotted on nitrocellulose-coated glass slides [3]. Slides were blocked with 1% BSA/phosphate-buffered saline/Tween (PBST). Sera were added to the arrays. After washing, anti-human immunoglobulin G (IgG) conjugated to Alexa Fluor 647 dye was added. Arrays were washed and dried (Partnership, Evry, France). Arrays were scanned with a GenePix 4000B Fluorescent Scanner, GenePix.Molecular Devices, Sunnyvale, California, USA. Data were acquired with GenePix Pro software and processed using ProtoArray Prospector 2.0 (Invitrogen). A panel of values was calculated for each protein array, including the *Z*-score, the Chebyshev inequality precision (CIP) value and the coefficient of variation (CV) value as previously described [3]. A *Z*-score >3.0, a CIP value <0.05 and a CV <0.5 define a positive spot.

### Sera samples for ELISA analysis

RA patients were chosen from the Rheumatology Ward at Hospital Sainte Marguerite, Marseille and from the Rheumatology Ward at Hospital Jean Minjoz, Besançon. AS patients and PsA patients were chosen from the Rheumatology Ward at Hospital Sainte Marguerite, Marseille. SLE patients were chosen from the Rheumatology Ward at Hospital Sainte Marguerite, Marseille and from the Internal medicine Ward at Hospital Bretonneau, Tours. Patients with SSc were from Hospitals Saint Antoine, Saint Louis, Paris; Hospital Claude Huriez, Lille and Hospital Sainte Marguerite, Marseille. Volunteers from the laboratory staff and the Marseille Blood Transfusion Center staff served as normal controls.

### Detection of autoantibodies by ELISA

Plates were coated with purified proteins and blocked with PBS containing 5% milk. Sera diluted to 1:100 in PBS were incubated for 3 h. After washing with 0.1% Tween 20, peroxydase-conjugated anti-human IgG (Sigma, Saint-Quentin Fallavier, France) was added. Optical density (OD) was read at 405 nm. Background OD was obtained by adding each serum to a well without protein. Positive sera were defined by an OD value more than twice the background OD [3]. Moreover, positive sera were defined by an OD value more than twice the mean OD of the control groups (AS, PsA, SLE, SSC and healthy subjects). Data were similar using both methods. *P*-values were calculated using the chi squared test.

### Synthetic peptides

15-mer peptides encompassing residues from WIBG (locus NM_032345.1) GABARAPL2 (locus NM_007285.5) and ZNF706 (locus NM_016096.1) overlapping on seven amino acids were synthesized using the solid-phase system, and purified (Neosystem, Strasbourg, France).

### Epitope mapping

Plates were coated overnight with 10 μg/well peptides diluted in PBS, pH 7.4. Plates were blocked with 5% milk PBS. Sera, diluted to 1:100 in PBS, were incubated for 2 h. After washing with 0.1% Tween 20, peroxydase-conjugated anti-human IgG, (Sigma, France) was added. OD was read at 405 nm. Background OD was obtained by adding each serum to a well without peptide. Positive sera were defined by an OD value more than twice the background OD. *P*-values were calculated using the chi squared test.

## Results and discussion

### Autoantibody pattern associated with RA patients and controls

We selected sera from 20 RA patients with disease duration less than 1 year and compared their reactivity pattern on protein arrays with that of sera from 19 RA patients with disease duration more than 5 years, and with sera from 23 controls. The control group included seven AS patients, two SLE patients, four SSc patients and ten healthy subjects. Autoantibodies were detected by anti-human IgG antibody.

The sera of RA patients with disease duration more than 5 years bound, on average, 101 proteins. AS sera bound 112 proteins, SLE sera bound 91 proteins, SSc sera bound 103 proteins and healthy controls' sera bound 89 proteins (data not shown). Sera from patients with early RA bound, on average, 58 proteins. Among these proteins, we identified 25 that were recognized by 30% to 60% of RA patients with disease duration less than 1 year and less than 10% of controls (Table 2). These proteins were recognized by 0% to 37% of RA patients with disease duration more than 5 years.

**Table 2:** Protein array analysis

				**Percentage of positive sera**		
**Protein**	**Protein name**	**Protein abbreviation**	**Reference**	**RA <1 year** **(20)**	**RA >5 years (19)**	**Controls (23)**
P1	IMMUNOGLOBULIN (CD79A) BINDING PROTEIN 1	IGBP1	NM_001551.1	60	0	0
P2	PEPTIDYL ARGININE DEIMINASE, TYPE IV	PAD4	NM_012387.1	45	37	0
P3	FK506 binding protein	FKBP3	NM_002013.2	45	5	10
P4	TROPOMYOSIN 2 (BETA)	TPM2	NM_003289.3	45	0	0
P5	ZINC FINGER PROTEIN 706	ZNF706	NM_016096.1	45	2	10
P6	COILED-COIL DOMAIN CONTAINING 72	CCDC72	NM_015933.1	40	0	0
P7	Hypothetical protein MGC17403 (MGC17403), Transcription elongation factor A (SII) N-terminal and central domain containing (TCEANC)	MGC17403	NM_152634.1	40	0	0
P8	ELG PROTEIN	C17orf85	NM_018553.1	40	0	0
P9	HYPOTHETICAL PROTEIN MGC11257	C7orf50	NM_032350.3	40	0	0
P10	T complex mouse like	TCP10L	NM_144659.1	40	5	5
P11	ELONGATION FACTOR 1 HOMOLOG (S. CEREVISIAE)	ELOF1	NM_032377.2	40	5	0
P12	FGF12	FGF12	NM_004113.3	40	0	0
P13	SYNAPTOTAGMIN I	SYT1	NM_005639.1	40	0	0
P14	WITHIN BGCN HOMOLOG (DROSOPHILA)	WIBG	NM_032345.1	40	10	0
P15	YY1 TRANSCRIPTION FACTOR	YY1	NM_003403.3	40	0	0
P16	Eucaryotic transl factor 1A	EIF1AX	NM_001412.2	40	5	5
P17	DOUBLECORTEX LISSENCEPHALY, X-LINKED (DOUBLECORTIN)	DCX	NM_178151.1	35	0	0
P18	LAMIN A/C	LMNA	BC033088.1	35	0	0
P19	TROPOMYOSIN 4	TPM4	BC002827.1	30	0	0
P20	ACIDIC (LEUCINE-RICH) NUCLEAR PHOSPHOPROTEIN 32 FAMILY, MEMBER E	ANP32E	NM_030920.1	30	0	0
P21	HYPOTHETICAL PROTEIN MGC20255	CCDC97	NM_052848.1	30	5	0
P22	SPANX-N3 PROTEIN	SPANXN3	NM_001009609.1	30	0	0
P23	THYMIC STROMAL LYMPHOPOIETIN	TSLP	NM_138551.1	30	0	0
P24	GABA(A) RECEPTOR-ASSOCIATED PROTEIN-LIKE 2	GABARAPL2	NM_007285.5	30	0	0
P25	SCY1like	SCYL1	BC009967.1	30	0	5

RA, rheumatoid arthritis.

### Autoantibodies specific for RA patients

**Figure 1:**
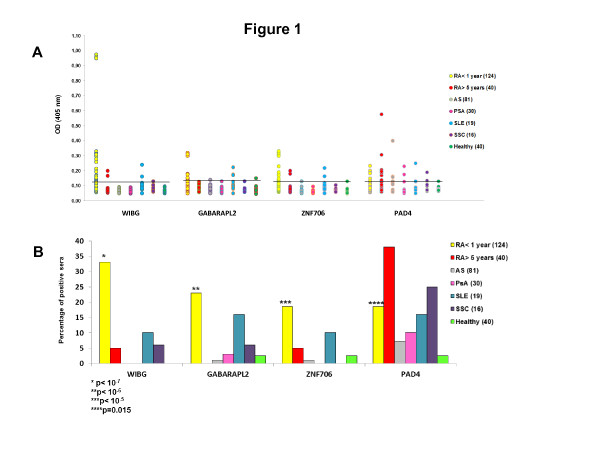
**Autoantibodies to within BGCN homolog (WIBG), GABA(A) receptor-associated protein-like 2 (GABARAPL2), zinc finger protein 706 (ZNF706) and peptidyl arginine deiminase 4 (PAD4) proteins are significantly detected in patients with early rheumatoid arthritis (RA)**. Sera from RA patients and controls were tested on the 25 purified proteins by ELISA. (**A**) Optical density (OD) read at 405 nm. The cutoff, marked as a horizontal line, defines positive sera with an OD value higher than twice the mean OD of the control groups (patients with ankylosing spondylitis (AS), psoriatic arthritis (PsA), systemic lupus erythematosus (SLE), systemic sclerosis (SSC) and healthy subjects). (**B**) Percentage of positive sera. *P-*values were obtained by comparing the group with early RA to the control groups (AS, PsA, SLE, SSC and healthy subjects).

To confirm the validity of protein array detection, we developed ELISAs using the 25 purified proteins. We tested sera from 124 RA patients with disease duration less than 1 year, 40 RA patients with disease duration more than 5 years, 81 AS patients, 30 PsA patients, 19 SLE patients, 16 SSC patients and 40 healthy subjects. Four proteins were significantly recognized by early RA patients and less than 10% of controls (Figure 1A,B). These proteins are WIBG, GABARAPL2, ZNF706 and PAD4 (peptidyl arginine deiminase 4).

Autoantibodies to GABARAPL2, ZNF706 and PAD4 were less sensitive and specific for early RA than autoantibodies to WIBG. Indeed, autoantibodies to PAD4 identified 38% of RA patients with disease duration more than 5 years. Moreover, autoantibodies to GABARAPL2, ZNF706 and PAD4 were also found in SLE and SSC patients. Autoantibodies to WIBG were strongly associated with early RA. Indeed, 33% of RA patients' sera recognized WIBG versus 5% of RA patients with disease duration more than 5 years (*P *= 0.0004), 0% of AS patients (*P* <10^-7^), 0% of PsA patients (*P *= 0.0002), 10% of SLE patients (*P *= 0.05), 6% of SSc patients (*P *= 0.03) and 0% of healthy individuals (*P *= 0.00003).

In combination, WIBG, GABARAPL2 and ZNF706 proteins identified 48% of patients with early RA versus 10% of RA patients with disease duration more than 5 years and 6% of controls (Table 3). Among patients with early AS, PsA, SLE and SSC with disease duration less than one year, only 2/34 were positive for GABARAPL2 and WIBG (data not shown). Of interest, the combination of WIBG, GABARAPL2 and ZNF706 proteins identified 43% of early RA patients negative for anti-CCP, 44% of early RA patients negative for RF and 39% of early RA patients negative for anti-CCP and RF.

**Table 3:** Autoantibodies to WIBG, GABARAPL2, ZNF706 and PAD4 proteins in patients with early RA

								WIBG, ZNF706 and GABARAPL2
		Anti-CCP	RF	PAD4	WIBG	ZNF706	GABARAPL2	combination
RA <1 year (*n *= 124) vs 186 controls (AS, PsA, SLE, SSc, and healthy)	Sensitivity (%)	70	68	18.5	33	18.5	23	48
	Specificity (%)	98	nd	91	98	98	96	94
	PPV (%)	96	nd	57	93	85	80	84
	NPV (%)	81	nd	63	69	64	65	73
								
RA <1 year negative for anti-CCP (*n *= 37) vs 186 controls (AS, PsA, SLE, SSc, and healthy)	Sensitivity (%)	0	24	11	16	11	27	43
	Specificity (%)	nd	nd	91	98	98	96	94
	PPV (%)	nd	nd	19	67	50	59	59
	NPV (%)	nd	nd	84	86	85	87	89
								
RA <1 year negative for RF (*n *= 39) vs 186 controls (AS, PsA, SLE, SSc, and healthy)	Sensitivity (%)	23	0	10	15	15	28	44
	Specificity (%)	98	nd	91	98	98	96	94
	PPV (%)	69	nd	19	67	60	61	61
	NPV (%)	84	nd	83	85	85	86	89
								
RA <1 year negative for anti-CCP and RF (*n *= 28) vs 186 controls (AS, PsA, SLE, SSc, and healthy)	Sensitivity (%)	0	0	4	7	11	32	39
	Specificity (%)	nd	nd	91	98	98	96	94
	PPV (%)	nd	nd	5	40	43	56	50
	NPV (%)	nd	nd	86	87	88	90	91

WIBG, within BGCN homolog (*Drosophila*); GABARAPL2, GABA(A) receptor-associated protein-like 2; ZNF706, zinc finger protein 706; PAD4, peptidyl arginine deiminase 4; RA: rheumatoid arthritis; CCP, cyclic citrullinated peptide; RF, rheumatoid factor; AS, ankylosing spondylitis; PsA, psoriatic arthritis; SLE, systemic lupus eryth ematosus; SSc, systemic sclerosis; PPV, positive predictive value; NPV, negative predictive value; nd, not determined.

### Epitope mapping on WIBG, GABARAPL2 and ZNF706

**Figure 2:**
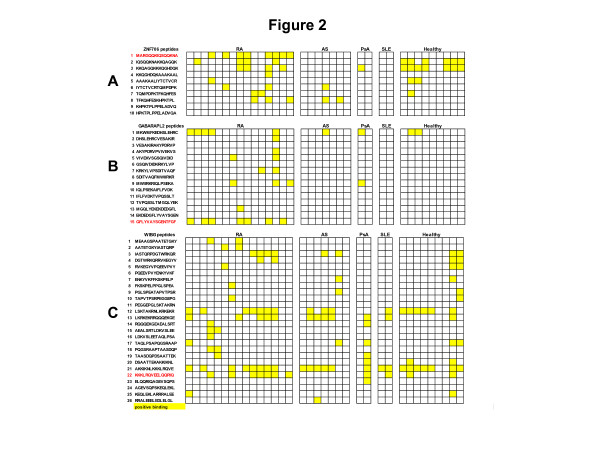
**Epitope mapping on zinc finger protein (706ZNF706), GABA(A) receptor-associated protein-like 2 (GABARAPL2) and within BGCN homolog (WIBG)**. Sera from patients with early RA, and from patients with ankylosing spondylitis (AS), psoriatic arthritis (PsA), systemic lupus erythematosus (SLE), and healthy controls were tested for binding to ZNF706, GABARAPL2 and WIBG peptides by ELISA. After washing, peroxydase-conjugated anti-human immunoglobulin G was added. Optical density (OD) was read at 405 nm. Background OD was obtained by adding each serum to a well without peptide. Positive sera were defined by an OD value higher than twice the background OD (yellow).

To identify B cell epitopes on ZNF706, GABARAPL2 and WIBG, we synthesized overlapping 15-mer peptides encompassing the entire sequence of these proteins. As a first step, we screened these peptides with the sera of 15 RA patients known to contain autoantibodies to ZNF706, GABARAPL2 and WIBG and 20 controls. Among the ten peptides from ZNF706, eight were recognized by at least one of the tested sera (Figure 2). One peptide, P1, was specifically recognized by sera from RA patients. Indeed, 8/15 RA sera recognized P1 versus 0/20 controls (Figure 2A).

Among the 15 peptides from GABARAPL2, 8 were recognized by at least one of the tested sera (Figure 2B). One peptide, P15, was specifically recognized by sera from RA patients. Indeed, 7/15 RA sera recognized P15 versus 0/20 controls.

Among the 26 peptides from WIBG, 18 were recognized by at least one of the tested sera (Figure 2C). One peptide, P22, was preferentially recognized by sera from RA patients. Indeed, 7/15 RA sera recognized P22 versus 4/20 controls.

To confirm these reactivities, we tested by ELISA sera from 60 patients with early RA and 106 controls (20 AS, 24 PsA and 22 SLE patients, and 40 healthy subjects) on ZNF706 P1, GABARAPL2 P15 and WIBG P22 (Table 4).

**Table 4:** Autoantibodies to WIBG, GABARAPL2, ZNF706 peptides in patients with early RA

							WIBG P22, ZNF706 P1 and GABARAPL2 P15 combination
		Anti-CCP	RF	WIBG P22	ZNF706 P1	GABARAPL2 P15	
RA <1 year (*n *= 60) vs 106 controls (AS, PsA, SLE, and healthy)	Sensitivity (%)	72	70	30	35	37	45
	Specificity (%)	100	nd	95	100	95	92
	PPV (%)	100	nd	78	100	81	75
	NPV (%)	86	nd	71	73	73	75
							
RA <1 year negative for anti-CCP (*n *= 17) vs 106 controls (AS, PsA, SLE, and healthy)	Sensitivity (%)	0	29	12	6	23	41
	Specificity (%)	nd	nd	95	100	95	92
	PPV (%)	nd	nd	18	10	31	44
	NPV (%)	nd	nd	87	86	88	91
							
RA <1 year negative for RF (*n *= 17) vs 106 controls (AS, PsA, SLE, and healthy)	Sensitivity (%)	29	0	12	12	18	35
	Specificity (%)	100	nd	95	100	95	92
	PPV (%)	100	nd	29	100	37	40
	NPV (%)	90	nd	87	88	88	90
							
RA <1 year negative for anti-CCP and RF (*n *= 12) vs 106 controls (AS, PsA, SLE, and healthy)	Sensitivity (%)	0	0	8	8	25	42
	Specificity (%)	nd	nd	95	100	95	92
	PPV (%)	nd	nd	17	100	37	36
	NPV (%)	nd	nd	90	91	92	93

WIBG, within BGCN homolog (*Drosophila*); GABARAPL2, GABA(A) receptor-associated protein-like 2; ZNF706, zinc finger protein 706; RA, rheumatoid arthritis; CCP, cyclic citrullinated peptide; RF, rheumatoid factor; AS, ankylosing spondylitis; PsA, psoriatic arthritis; SLE, systemic lupus eryth ematosus; SSc, systemic sclerosis; PPV, positive predictive value; NPV, negative predictive value; nd, not determined.

Autoantibodies to ZNF706 P1, GABARAPL2 P15 and WIBG P22 were respectively found in 35%, 37% and 30% of patients with early RA, with a specificity ranging from 95% to 100%. In combination, these three peptides identified 45% of patients with early RA versus 8% of controls (*P *<10^-7 ^for 60 patients with early RA versus 106 controls). Of interest, the combination of these peptides identified 41% of early RA patients negative for anti-CCP, 35% of early RA patients negative for RF and 42% of early RA patients negative for anti CCP and RF.

## Discussion

To get an insight into the early immunological events leading to the development of RA and to develop diagnostic tools for early RA, we screened 8,268 protein arrays with sera from patients with early RA (less than one year) RA with disease duration more than 5 years, and controls. We selected 25 proteins that could constitute specific serologic markers of early RA because they identified 30% to 60% of patients with early RA and less than 10% of controls.

To confirm the validity of protein array detection, we used the same proteins in ELISAs to screen more sera from patients and controls. Among these proteins, only one, PAD4, the enzyme that converts arginine into citrulline, was already known to be a target for RA autoantibodies [4-8]. Autoantibodies to PAD4 are more frequent in established than in early RA. In a previous study, we have identified peptide targets of anti-PAD4 autoantibodies [8]. Most anti-PAD4 positive sera recognized peptides located both in the N-terminal domain (amino acids 211 to 290) and the C-terminal domain (amino acids 601 to 650) of PAD4. Peptide recognition in the substrate-binding domain of PAD4 influences the enzymatic activity of PAD4. Indeed, most autoantibodies to PAD4 inhibit PAD4-mediated citrullination.

By using protein arrays and ELISA assays, we validated three new autoantibodies significantly associated with early RA. These autoantibodies recognize ZNF706, GABARAPL2, and WIBG. ZNF706 belongs to the C2H2-type zinc-finger protein family (Additional file 1: Table S1) [9]. GABARAPL2 is involved in autophagy, the process by which proteins and organelles are sequestered in autophagosomal vesicles and delivered to the lysosome for degradation [10]. WIBG is a ribosome-associated protein involved in the disassembly of exon junction complexes (EJCs). EJCs, assembled during mRNA splicing, transport mRNA during nuclear export into the cytoplasm and are removed during translation [11]. WIBG enhances translation of mRNA. WIBG has also been shown to enhance the translation of viral genes, acting as a so-called chaperone.

WIBG is the protein recognized by the highest number of sera from patients with early RA. The percentage of anti-WIBG-positive RA patients decreases with disease duration. Indeed, WIBG is recognized by 33% of RA patients with disease duration less than 1 year, 12% of RA patients with disease duration between 1 and 5 years and only 5% of RA patients with disease duration more than 5 years (data not shown).

The level of many RA-associated autoantibodies is already known to decrease with time. For example, anti-nuclear antibodies (ANA) are positive in 20% to 30% of patients with early RA. However, a few months after disease onset, the ANA test may turn and remain negative [12]. This is also true for anti-Sa and RF [13].

Disease-modifying therapies could explain the autoantibody decrease in RA patients. Alternatively, the early recognition of autoantigens in RA could give an insight into the early mechanisms that lead to disease development. In this respect, it is remarkable that proteins associated with DNA or RNA are often initial targets of immune responses. Later on, disease development is accompanied by narrowing of the immune response to specific targets. The best example of this phenomenon is the evolution of Sharp's syndrome into RA, scleroderma or lupus, which is accompanied by loss of anti-ribonucleoprotein (RNP) antibodies and development of disease-specific autoantibodies [14]. Of interest, the combination of autoantibodies to WIBG, GABARAPL2 and ZNF706 may be used to help diagnosis of early RA, especially in patients without anti-cyclic citrillunated protein (CCP) antibodies and RF.

Finally, we analyzed the epitopes recognized by autoantibodies to ZNF706, GABARAPL2, and WIBG in a direct ELISA, using a set of synthetic peptides and sera from patients with early RA. Fine epitope mapping on ZNF706, GABARAPL2, and WIBG enabled us to identify three linear peptides that may prove interesting diagnostic tools in the early stages of RA. The next step will be to include more patients and controls to identify the best marker combination for diagnosis of early RA.

## Conclusions

There is a great need for new biological markers of RA to permit early intervention to potentially prevent inflammation and joint destruction. To identify new autoantibody signatures in RA patients, we have analyzed autoantibodies in the sera of patients with early RA (duration less than one year) on protein arrays containing 8,000 human proteins. We found and validated three new autoantigens significantly associated with early RA. These autoantigens (ZNF706, GABARAPL2 and WIBG) and peptides derived from these autoantigens can be used to identify RA at the early stage of the disease.

## List of abbreviations

ACPA: anti-citrullinated protein antibodies; ACR: American College of Rheumatology; ANA: anti-nuclear antibodies; AS: ankylosing spondylitis; BSA: bovine serum albumin; CIP: Chebyshev inequality precision; CV: coefficient of variation; EJC: exon junction complexes; ELISA: enzyme-linked immunosorbent assay; EULAR: European League Against Rheumatism; GABARAPL2: GABA(A) receptor-associated protein-like 2; GST: glutathione-S-transferase; IgG: immunoglobulin G; OD: optical density; PAD4: peptidyl arginine deiminase 4; PBS: phosphate-buffered saline; PBST: phosphate-buffered saline/Tween; PsA: psoriatic arthritis; RA: rheumatoid arthritis; RF: rheumatoid factor; RNP: ribonucleoprotein; SLE: systemic lupus erythematosus; SSc: systemic sclerosis; WIBG: within BGCN homolog (*Drosophila*); ZNF706: zinc finger protein 706.

## Conflicts of interest

A patent was submitted by INSERM TRANSFERT in May 2011, submission number: 11305584; Receiving Office, European Patent Office, The Hague.

## Authors' contributions

IA and JR contributed to the conception and design of the study and drafted the manuscript. CC, ET, NL, NB, DW, ED and JR collected clinical samples and data. CC, FA, MM and IA performed the experiments. All authors were involved in the acquisition of data and the revision of the manuscript. All authors read and approved the final manuscript.

## Supplementary Material

Additional file 1 **Table S1 Characteristics of the early RA autoantigens**. Description of early autoantigens (WIBG, ZNF706 and GABARAPL2 proteins). Click here for file
