# Supplementary material for: New autoantibodies in early rheumatoid arthritis
Source: Arthritis Res Ther. 2013 Jul 25;15(4):R78. doi: 10.1186/ar4255 (PMC3978570; doi:10.1186/ar4255)
Supplement: Additional file 1 — Table S1 Characteristics of the early RA autoantigens. Description of early autoantigens (WIBG, ZNF706 and GABARAPL2 proteins). [file ar4255-S1.PDF]

Table S1: Characteristics of the early RA autoantigens

	Protein names	Gene names	Functions	Biological process	Subcellular location	Domain	Sequence similarities
**WIBG**	WITHIN BGCN homolog (Drosophila)<br>Partner of Y14 and mago<br>Protein wibg homolog	WIBG<br>PYM	Ribonucleoprotein	Disassembly of exon junction complexes<br>Translation regulation<br>Nuclear cytoplasmic shuttle	Cytoplasm<br>Nucleus	EIF2A-like region<br>Coiled coil	Wibg family
**GABARAPL2**	Gamma-aminobutyric acid receptor-associated protein-like 2<br>GABA(A) receptor-associated protein-like 2<br>Ganglioside expression factor 2<br>Short name=GEF-2<br>General protein transport factor p16<br>Golgi-associated ATPase enhancer of 16 kDa<br>Short name=GATE-16<br>MAP1 light chain 3-related protein	GABARAPL2<br>FLC3A<br>GEF2	ATPase binding<br>GABA receptor binding<br>SNARE binding<br>Beta-tubulin binding<br>Microtubule binding	Autophagy<br>Intra-Golgi vesicle-mediated transport<br>Positive regulation of ATPase activity<br>Protein transport	Golgi apparatus		MAP1 LC3 family
**ZNF706**	Zinc finger protein 706	ZNF706<br>HSPC038<br>PNAS-113	Zinc ion binding	Transcriptional activation or inhibition	Intracellular	C2H2-type zinc finger<br>DNA binding domain	
